# Mental Health of Children and Adolescents Amidst COVID-19 and Past Pandemics: A Rapid Systematic Review

**DOI:** 10.3390/ijerph18073432

**Published:** 2021-03-26

**Authors:** Salima Meherali, Neelam Punjani, Samantha Louie-Poon, Komal Abdul Rahim, Jai K. Das, Rehana A. Salam, Zohra S. Lassi

**Affiliations:** 1 Faculty of Nursing, University of Alberta, Edmonton, AB T6G 1C9, Canada; npunjani@ualberta.ca (N.P.); slouiepo@ualberta.ca (S.L.-P.); 2 Aga Khan University Hospital, Karachi City, Sindh 74800, Pakistan; komalrahim44@gmail.com (K.A.R.); jai.das@aku.edu (J.K.D.); rehana.salam@aku.edu (R.A.S.); 3 Robinson Research Institute, University of Adelaide, Adelaide, SA 5005, Australia; zohra.lassi@adelaide.edu.au

**Keywords:** COVID-19, pandemic, epidemic, mental health, children, adolescents

## Abstract

*Background:* The COVID‑19 pandemic and associated public health measures have disrupted the lives of people around the world. It is already evident that the direct and indirect psychological and social effects of the COVID‑19 pandemic are insidious and affect the mental health of young children and adolescents now and will in the future. The aim and objectives of this knowledge-synthesis study were to identify the impact of the pandemic on children’s and adolescent’s mental health and to evaluate the effectiveness of different interventions employed during previous and the current pandemic to promote children’s and adolescents’ mental health. *Methodology:* We conducted the systematic review according to the Preferred Reporting Items for Systematic Reviews and Meta-Analyses (PRISMA) guidelines and included experimental randomized and nonrandomized controlled trials, observational studies, and qualitative studies. *Results*: Of the 5828 articles that we retrieved, 18 articles met the inclusion criteria. We thematically analyzed them and put the major findings under the thematic areas of impact of the pandemic on children’s and adolescents’ mental health. These studies reported that pandemics cause stress, worry, helplessness, and social and risky behavioral problems among children and adolescents (e.g., substance abuse, suicide, relationship problems, academic issues, and absenteeism from work). Interventions such as art-based programs, support services, and clinician-led mental health and psychosocial services effectively decrease mental health issues among children and adolescents. *Conclusion:* Children and adolescents are more likely to experience high rates of depression and anxiety during and after a pandemic. It is critical that future researchers explore effective mental health strategies that are tailored to the needs of children and adolescents. Explorations of effective channels regarding the development and delivery of evidenced-based, age-appropriate services are vital to lessen the effects and improve long-term capacities for mental health services for children and adolescents. *Key Practitioner Message*: The COVID-19 pandemic’s physical restrictions and social distancing measures have affected each and every domain of life. Although the number of children and adolescents affected by the disease is small, the disease and the containment measures such as social distancing, school closure, and isolation have negatively impacted the mental health and well-being of children and adolescents. The impact of COVID-19 on the mental health of children and adolescents is of great concern. Anxiety, depression, disturbances in sleep and appetite, as well as impairment in social interactions are the most common presentations. It has been indicated that compared to adults, this pandemic may continue to have increased long term adverse consequences on children’s and adolescents’ mental health. As the pandemic continues, it is important to monitor the impact on children’s and adolescents’ mental health status and how to help them to improve their mental health outcomes in the time of the current or future pandemics.

## 1. Introduction

COVID‑19 has impacted the lives of people around the world. During times of uncertainty, it is crucial to focus on mental and emotional well-being to navigate these difficult circumstances in a healthy way. In the absence of a vaccine, public health measures have been implemented to prevent and reduce the spread of the virus. Schools have been closed, and most extracurricular activities for children and adolescents that typically occur outside the home and in group settings have been cancelled. Children and adolescents are experiencing a prolonged state of physical isolation from their peers, teachers, extended family, and community networks. Social distancing and school closures therefore increase the mental health problems of children and adolescents, who are already at higher risk for developing mental health problems compared to adults at a time when they are also experiencing anxiety over a health threat and threats to family employment/income.

School routines are essential coping mechanisms for young people with mental health issues. During school closures, children lose an anchor in life, and their symptoms can return. For children and adolescents with mental health issues, school closures mean a lack of access to the resources that they usually have through schools [1]. In a survey that the mental health charity Young Minds conducted, over 2000 children and adolescents in the UK verbalized that the pandemic has made their mental health conditions worse and that they are unable to access mental health support. Peer support groups, face-to-face services, and telephone support services can be challenging for some children [2].

The responses of children and adolescents to a crisis situation depends upon their prior exposure to emergency situations, physical and mental health issues, the socioeconomic circumstances of the family, and their cultural background [1,3]. Different studies have shown that crisis events negatively impact the psychological well-being of children and adolescents [4,5,6]. A recent study that Jiao et al. (2020) conducted in China screened children and adolescents for behavioral and emotional distress due to the COVID‑19 pandemic. Anxiety, depression, distraction, irritability, and the fear that family members would contract the deadly disease were the most common problems that they identified [7]. Moreover, when adolescents struggle with emotional problems, they often turn to drug use to help them to manage painful or difficult feelings [3]. The mechanisms that they use to cope with stressful situations is not distinct from those of adults; however, because children’s and adolescents’ brains are still developing, the consequences of “self-medication” are more instantaneously challenging [8,9]. In the short term, substance use can help to relieve undesirable mental health symptoms such as hopelessness, anxiety, irritability, and negative thoughts. However, in the longer term, it aggravates them and often ends in abuse or dependence.

In April 2020, the United Nations International Children’s Emergency Fund (UNICEF) joined some partner organizations and conducted a survey on 1700 children and adolescents from 104 countries to understand how the COVID‑19 pandemic was affecting their lives, particularly their mental health and psychological well-being. Children and adolescents reported high levels of stress, which can affect their brain development, sometimes with irreparable long-term consequences [10]. As a result, many organizations have developed online tools and targeted interventions to improve the mental health of young children during COVID‑19. For example, the World Health Organization (WHO) [11] has developed a tool called “Helping Children Cope With Stress During the 2019-nCoV Outbreak”. Similarly, many organizations around the globe have initiated telephone and text-messaging services to support children with mental health issues and those who are undergoing crises such as substance abuse during the pandemic (Takeuchi, 2020). However, many questions remain: Which interventions work? How are they designed, implemented, and evaluated? Are these interventions sustainable and potentially scalable?

Although some research exists on the psychological impact of Severe Acute Respiratory Syndrome on patients and health-care workers, not much is known about the effects on children and adolescents. In addition, COVID‑19 is much more widespread than Severe Acute Respiratory Syndrome and other epidemics were on a global scale. As the pandemic continues, it is important to monitor the impact on children’s and adolescents’ mental health and status and improve their mental health outcomes in the current and future pandemics. A rapid systematic review was conducted to provide timely evidence synthesis to inform urgent healthcare policy decision-making. A rapid review adheres to the essential principles of systematic reviews, including scientific rigor, transparency, and reproducibility [12,13]. The aim of this rapid systematic review study was to identify and evaluate (a) the impact of the pandemic/epidemic on children’s and adolescents’ mental health, (b) the effectiveness of different interventions employed during the current and previous pandemics to promote children’s and adolescents’ mental health, and (c) to identify knowledge gaps in these contexts. This rapid review aims to advise public health and policy makers on strategies and interventions to improve mental health among children and adolescents in pandemics.

## 2. Methods

This review was conducted in accordance to the Preferred Reporting Items for Systematic Reviews and Meta-Analyses (PRISMA) guidelines (Moher, Liberati, Tetzlaff, and Altman, 2009) and included experimental studies (randomized—individually or cluster—and nonrandomized controlled trials), observational studies with an internal comparison group (cohort—prospective and retrospective—and case-control) studies, and qualitative studies. Case reports, case studies, opinions, editorials, commentaries, letters, conference abstracts, and reviews or systematic reviews were excluded. The protocol of this systematic review was registered with PROSPERO (protocol ID: CRD42020223750).

### 2.1. Search Strategy

Working with a research librarian from the University of Alberta, we searched MEDLINE, EMBASE, Web of Science Index Medicus, CINAHL, Lilacs, CENTRAL (Cochrane Library), eLENA (WHO), and the WHO COVID-19 databases. Nonindexed databases, including Google Scholar, and the preprint databases MedRxiv [14] and ChinaXiv [15] were also searched. To identify missing papers, bibliographies of all included studies and all relevant systematic reviews were hand-searched. Restrictions on language and date were not utilized. Search results were uploaded to COVIDENCE following the removal of duplicates (see Supplementary Material File S1 for detailed search strategy).

### 2.2. Types of Participants

Studies were included if they were conducted on school-age children and adolescents (5 to 19 years) in low-middle and high income countries. Studies that involved broader age groups were included provided that they had subgroup data for 5- to 19-year-olds. Studies were excluded if participants specifically involved children and adolescents with mental health diseases, those who are homeless, and those with substance abuse issues.

### 2.3. Type of Exposure and Interventions

Studies on recent COVID‑19 and past pandemics were included (e.g., Severe Acute Respiratory Syndrome Coronavirus, H1N1 influenza, equine influenza, Ebola, Middle East Respiratory Syndrome-Related Coronavirus). Additionally, studies were included focusing on interventions delivered to school-age children, adolescents, and their families to improve their mental health.

### 2.4. Types of Outcomes

The primary outcomes include anxiety and depression, and the secondary outcomes include fear of infection, frustration, boredom, fear of pandemic-related uncertainty, fear of running out of basic supplies, and finances. Knowledge gaps were identified, along with the applicability of the findings of the current pandemic to improve the mental health of school-aged children and adolescents.

### 2.5. Study Selection

Articles were included if the study exclusively examined the impact of COVID-19 and past pandemics/epidemics on children’s and adolescents’ mental health. Detailed inclusion and exclusion criteria are shown in Table 1. Using Covidence, a Web-based tool that helps to identify studies and involves data-extraction processes [16], two reviewers (NP and SM) independently screened all potential articles. In case of disagreement, both reviewers read the paper and discussed it until a consensus was reached. Following, independent screening of the full texts of eligible articles was conducted and papers that satisfied all of the following inclusion criteria were included in this review.

### 2.6. Data Extraction

Relevant data were extracted from each study, including the year and country of publication, the study design, the target population, pandemic exposure, interventions, and the outcomes that the researchers measured (see Table 2). One reviewer (NP) used a form that the research team had developed to extract data. A second reviewer (SM) verified all of the data extraction and checked for accuracy and completeness. Disagreements were resolved through discussion.

### 2.7. Quality Assessment

The methodological quality of the studies was assessed by using the Mixed Methods Appraisal Tool. It appraises the methodological quality of five categories of studies: qualitative research, randomized controlled trials, nonrandomized studies, quantitative descriptive studies, and mixed-methods studies [35].

### 2.8. Data Analysis/Synthesis

A tailored approach to synthesis the data was employed, using an evidence table to report the key components of each study (Table 2). Data were aggregated and analyzed according to the study’s outcomes and objectives. Descriptive (narrative) analyses of the included studies were conducted. Due to the heterogeneity of the papers, such as in the study design, the type of pandemic, the age groups of the participants, and the reported outcomes, conducting a meta-analysis was not possible. Therefore, findings were synthesized according to the type of pandemic, the reported outcomes, and the study design.

## 3. Results

**Figure 1:**
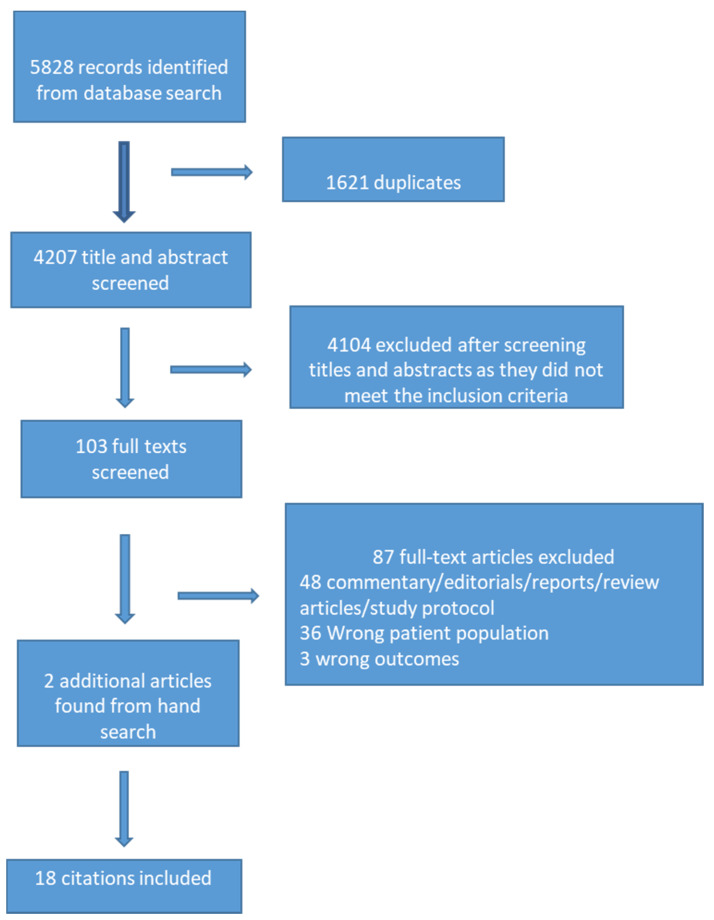
Preferred Reporting Items for Systematic Reviews and Meta-Analyses (PRISMA) diagram.

The search identified 5828 articles; we removed 1621 duplicates, which left 4207 unique citations. We excluded articles based on title/abstract screening (*n* = 4104). The majority of these articles pertained entirely to adults or university students over the age of 18. We retrieved 103 full-text articles, 16 of which met the inclusion criteria. We identified an additional two articles by scanning the reference lists of the included articles and added them, for a total of 18 articles (Figure 1).

### 3.1. Study Characteristics

Characteristics of the included studies are shown in Table 1. The included studies were conducted in four different pandemic/epidemic periods globally: Ebola (*n* = 3) [17,18,19], Equine Influenza (*n* = 1) [20], H1N1 (*n* = 1) [21], and COVID‑19 (*n* = 13) [22,23,24,25,26,27,28,29,30,31,32,33,34]. Most of the studies (*n* = 14) were cross-sectional [20,22,23,24,25,26,27,28,29,30,31,32,33,34], two were interventional [17,19], one was qualitative [18], and one was a mixed-method [21] study. The population age range varied between 5 and 19 years, inducing both boys and girls. The majority of the COVID‑19 studies (*n* = 9) were conducted in China [18,20,21,23,24,26,27,28,29,31,32,33,34] and the remaining four in Canada [25], India [30], Italy [22], and the United States [29]. The study on the Ebola pandemic was conducted in West Africa, the H1N1 epidemic study was conducted in the United States and Canada, and the study on equine influenza was conducted in Australia. The sample sizes ranged from 22 to 8079 subjects (mean = 1238) in the quantitative and intervention studies.

### 3.2. Methodological Quality

We assessed the methodological quality of the quantitative and qualitative articles by using the Mixed-Method Appraisal Tool. The qualitative researchers comprehensively discussed their methodologies, including the data-collection methods: the findings from the data were adequate, their interpretation of the results was sufficient, and data sources, collection, analysis, and interpretation were coherent [18]. We divided the studies that involved mixed methods into two components: qualitative and quantitative [21]. The qualitative researchers discussed their data collection and analysis method; however, the results were not transparent [17,22]. In contrast, intervention studies reported the representativeness of the data confounders, type of interventions, and outcomes [18,35]. On the whole, the mixed-method studies did not discuss in depth their interpretation of the data and failed to identify discrepancies [21]. The cross-sectional studies included some in which the researchers did not explicitly identify their inclusion and exclusion criteria [22,23]. Buzzi et al. (2020) did not test the reliability and validity of their instruments, and some researchers did not take into consideration other variables, inclusive of the confounders [26,31,32,34]. The included studies also demonstrated selection bias [29,31] and gender and demographic bias, which limited the generalizability of the findings [20]. In the interventional studies, the number of people who dropped out of research was fairly high, and required information on the compliance with the intervention was missing [17,19].

## 4. Study Findings

We thematically analyzed and put the major findings under the thematic areas of impact of pandemics on children’s and adolescents’ mental health. The studies included were categorized under four headings: the impact of the pandemic/epidemic on children’s and adolescents’ mental health, the impact of control measures on children’s and adolescents’ mental health, the impact of pandemic-/epidemic-related stigma on mental health, and the effectiveness of different interventions employed during the current and previous pandemics to promote children’s and adolescents’ mental health. A summary of the studies included in this rapid systematic review is presented in Table 1.

### 4.1. Impact of the Pandemic on Children’s and Adolescents’ Mental Health

Sixteen of the 18 studies examined the impact of the pandemic on children’s and adolescents’ mental health [18,20,21,22,23,24,25,26,27,28,29,30,31,32,33,34]. The majority of the researchers of the studies (*n* = 13) administered cross-sectional online surveys to evaluate the impact of the COVID-19 pandemic on children’s and adolescents’ mental health. In their cross-sectional survey, Taylor et al. (2008) evaluated the factors that influenced psychological distress during the equine influenza [20]. Sprang and Silman (2013) used a mixed-method design to investigate children’s psychosocial responses to the H1N1 pandemic, and in their qualitative study [21], Denis-Ramirez et al. (2017) explored the psychosocial impact of the Ebola epidemic in the daily lives of children in low-resource communities [18]. The most-reported outcome in these studies was the negative impact of a pandemic/epidemic on psychological health, which the researchers measured as anxiety, depression, fear, stigma, and posttraumatic stress symptoms. These studies reported that pandemics cause stress, worry, helplessness, and social and risky behavioral problems among children and adolescents (e.g., substance abuse, suicide, relationship issues, academic issues, absenteeism from work). Studies on the COVID‑19 pandemic reported that the current pandemic has significantly affected the emotional and behavioral experience of children and adolescents [22,23,24,25,26,27,28,29,30,31,32,33,34]. Depression and anxiety were higher among children and adolescents in other pandemics, and specifically COVID‑19 [22,23,24,25,26,27,28,29,30,31,32,33,34]. The researchers also reported that age, gender, knowledge about COVID‑19, degree of worry about epidemiological infection, and confidence about overcoming the outbreak significantly influenced the participants’ psychological status [28]. Studies also reported that female adolescents showed higher depression and anxiety levels during COVID‑19 than male adolescents did [23,24,25,32,33].

Studies (*n* = 4) also reported that the anxiety levels among the adolescent population were significantly higher than those in children [23,27,28,33]. In addition, adolescents in senior high school had the greatest depressive and anxiety symptoms [20,23,27,33], Liang et al.’s (2020) cross-sectional survey showed that the mental health of adolescents and youths is significantly related to being less educated (OR: = 8.71, 95%, CI: 1.97–38.43), the use of negative coping styles (OR: = 1.03, 95%, CI: 1.00–1.07), suffering from posttraumatic stress disorder (OR: = 1.05, 95%, CI: 1.03–1.07), and working as an employee (OR: = 2.36, 95%, CI: 1.09–5.09). Wang et al. (2020) reported that physical symptoms (myalgia and dizziness) and low evaluation of self-health status have a great impact on psychological well-being during the COVID‑19 pandemic, with a *p*-value of <0.05 [32]. Tian et al. (2020) included 1060 respondents, of whom more than 70% displayed moderate to high levels of psychological suffering such as phobic anxiety, obsessive-compulsive disorder, interpersonal sensitivity, and psychoticism [31]. Taylor et al. (2008) revealed that those who were living in red zones, which were highly infectious zones during the equine influenza (OR: = 2.00; 95%, CI: 1.57–2.55), and the amber or buffer zones (OR: = 1.83; 95% CI: 1.36–2.46) were more prone to psychological distress than those who lived in white zones, which were unaffected [20].

### 4.2. Impact of Control Measures to Contain the Effect on Children’s and Adolescents’ Mental Health

Seven studies reported that lifestyle transformation such as school closure, physical distancing, quarantine, isolation, and the threat of being infected is associated with depression and anxiety disorders among children and adolescents [18,21,24,25,29,30,34]. The psychological distress of fear, helplessness, worry, anxiety-related insomnia, isolation, boredom, and sadness was more common in the quarantine group [30]. These findings indicate that pandemic disasters, the subsequent disease-control measures, and containment responses can create conditions that families and children find traumatic. Sprang and Silman (2013) measures posttraumatic stress disorder using Posttraumatic Stress Disorder Reaction Index (PTSD-RI), and the PTSD Check List among children who experienced social distancing measures during the H1N1 pandemic [21]. The study found posttraumatic stress disorder in up to 30% of the children who were quarantined compared to those who had not been in isolation or quarantine during the H1N1 pandemic (1.1%; x^2^ = 49.56, *p* < 0.001) [21].

Ellis et al. (2020) used a regression model to interpret their findings and revealed that depression in female adolescents was significantly higher (β 0.15, *p* < 0.001) and that loneliness was also significantly more prevalent in female adolescents (β 0.08, *p* < 0.001) [25]. Researchers also reported that during the epidemic children and adolescents have spent more time online or on social media than they did before the crisis [24,25]. Ellis et al. found that the association between COVID‑19 stress and depression was strongest among adolescents who reported the highest social media use after the pandemic (β 0.96, *p* < 0.001) compared to adolescents with lower and average use [25]. The respondents in Ellis et al.’s (2020) and Duan et al.’s (2020) studies reported that adolescents spent 5–10 h per day online, which is a potential risk factor for addiction to the Internet or smartphone [24,25]. Duan et al. (2020) noted that smartphone addiction (OR 1.41, 95% CI: 1.10–1.18) and Internet addiction (OR 1.84, 95% CI: 1.21–2.81) are significantly associated with clinical depressive symptoms among children and adolescents [24].

### 4.3. Impact of Pandemic/Epidemic Related Stigma on Mental Health

In their qualitative study, Denis-Ramirez et al. (2017) reported the psychological impact of stigma in children who were orphaned because of the Ebola epidemic: They were excluded from social interactions because of their association with Ebola. The prevailing fear and stigma of Ebola undermined the willingness of community members to help orphaned children, and it had severe psychological repercussions for children orphaned by Ebola. Many of the children who participated in the study depicted orphaned children as ostracized, discriminated against, and labeled as “Ebola children.” The psychological effects of the stigma include the feeling of being alone, frustration, worry or sadness, rejection, and exclusion by family, friends, and communities [18].

### 4.4. Interventions Employed during the Previous and Current Pandemic to Promote Children’s and Adolescents’ Mental Health

We found only two studies on interventions to promote the mental health of children and adolescents during previous epidemics [17,19]. Decosimo et al. (2019) implemented a community psychosocial program with the aim of improving the mental health capacity of children aged 3–18 years during the Ebola epidemic [17]. The children received the interventions in settings where childhood trauma was prevalent. These interventions included expressive-art therapies, yoga therapy, and play therapy to help children to build healthy relationships, teach them child-specific trauma-coping skills, and build a safe space for children to express themselves. Of the 40 chosen sites, the children at 24 received a five-month intervention (Treatment Group 1), whereas at the remaining 16 sites they received three months of intervention (Treatment Group 2). Decosimo et al. (2019) found a statistically significant difference in the mean scores of 0.06 in treatment group 1 and 0.11 in treatment group 2, with a *p*-value of <0.001 pre- and postintervention. They also found a significant difference in the psychological stress symptoms over time before and after the children received the intervention. The findings indicate that the longer programming did not differ statistically from the shorter programming [17].

Kamara et al. (2017) focused on the mental health of children and adolescents aged 0–17 years during the Ebola virus epidemic in Sierra Leone, when the psychiatric hospital was closed to admissions to prevent disease transmission [19]. To provide mental health services in Sierra Leone, Kamara et al. (2017) established a nurse-led mental health and psychosocial support service at one of the largest government hospitals in the country, which had approximately 300 beds. To equip the nurses to provide mental health services, the WHO, CBM International, and local partners gave them psychological first-aid training focused on supporting those affected by the Ebola virus disease. These nurses prescribed medications in collaboration with physicians, and the children received counseling and a problem-solving approach to deal with their psychological suffering. The most commonly diagnosed mental health problems among the children and adolescents included distress, anxiety, depression, grief, and other social problems. Kamara et al. (2017) found that training nurses to manage mental health issues is an effective measure to strengthen the local capacity [19].

## 5. Discussion

Our findings from this current review shed light on the significant impact of pandemics on the mental health of children and adolescents. The results are overwhelming and demonstrate that pandemics are precursors to mental health decline [18,20,21,22,23,24,25,26,27,28,29,30,31,32,33,34]. Specifically, impairments in mental health leave children and adolescents with increased emotional stress, feelings of helplessness, and fear, which can evolve into mental illnesses such as anxiety, depression, and posttraumatic stress symptoms [18,20,21,22,23,24,25,26,27,28,29,30,31,32,33,34]. The consequences of the decline in mental health in young populations lead to engagement in unhealthy behaviors such as substance abuse, absenteeism from work, and school interruptions [22,24,27,28,33]. These results demonstrate that pandemics markedly contribute to a wide breadth of negative mental health consequences for children and adolescents, which establishes the critical need to explore effective strategies to promote positive mental health during pandemics.

The current global pandemic has underscored that attention on child and adolescent mental health is critical, indicating that timely measures are currently warranted during this COVID-19 era [22,23,24,25,26,27,28,29,30,31,32,33,34]. Despite the evidence that declining mental health status during the COVID‑19 pandemic is not unique to this population, this review illuminates that age-specific coping strategies are necessary to target the distinct needs of children and adolescents. Decosimo et al. (2019) revealed the positive impacts of age-specific mental health-promoting interventions during the Ebola pandemic: art therapies, yoga theory, play therapy, and collaboration with child life specialists. Although these interventions have revealed a significant difference in the psychological stress symptoms in children [17], it is critical that researchers explore alternative strategies to improve mental health outcomes during the current COVID‑19 pandemic.

The current global pandemic has resulted in a significant increase in the number of hours that children and adolescents spend online and on social media [24,25]. Studies have shown that smartphone/internet overuse can lead to mental or behavioral problems, cause poor studying performance, decrease real-life social interactions, result in neglect of their personal lives, and cause relationship disorders and mood dysfunction [36,37]. Despite the host of negative mental health outcomes for children and adolescents from the overuse of the Internet during a pandemic, as we reported earlier, there are limited age-specific and feasible interventions to combat this. Specifically, it is imperative that we target coping strategies for children and adolescents within the COVID‑19 pandemic measures (e.g., social distancing, school closures) and resource constraints (e.g., the capacity of parents, healthcare providers, and child life specialists) to optimize the feasibility of current mental health interventions. Mental health coping strategies should focus on measures that can be modified, such as innovative and age-appropriate research materials for children and adolescents.

Given the results that demonstrate that mental health decline is linked to education [26] and knowledge on COVID‑19 [28], developing educational materials is a unique opportunity to improve the mental health status of young populations. Translating research-informed evidence into educational materials will help to synthesize and tailor reliable information to the needs of its end-users [38]. In the case of younger populations, developing age-appropriate research-informed educational resources (such as videos, infographics, and comic books) can offer solutions to decrease adolescents’ mental health decline by improving their knowledge on pandemics. Keeping children and adolescents well informed through these modifiable strategies will target fear, worry, and stress about the unknown. As a result, it will alleviate the psychological distress and associated risky social and behavioral problems of children and adolescents, as we reported earlier [23,24,26,27,30,32]. Because depression and anxiety disorders are associated with the closure of schools, physical-distancing measures, and the increased time that children and adolescents spend online during pandemics [18,21,24,25,29,30,34], delivering informative educational materials through online media will give adolescents an opportunity to utilize their online hours productively by staying well-informed.

However, the negative mental health impacts of pandemics are not equally dispersed among this population. Notable are contributing factors such as identified gender, age, and levels of education. Because female adolescents have a higher risk of depression and anxiety during the COVID‑19 pandemic [23,24,25,32,33], generating mental health-promoting strategies specific to these gender and age categories is critical. Additionally, the development of mental health-coping strategies that integrate the education levels [26] at the various ages of children and adolescents warrants further exploration. Therefore, expanding research on strategies that are adaptable to individualized needs according to the gender, age, and education stratifications is fundamental to promote children’s and adolescents’ positive mental health status in the context of the COVID‑19 pandemic.

The majority of studies that we reviewed have revealed the impact of pandemics on the decline of children’s and adolescents’ mental health [18,20,21,22,23,24,25,26,27,28,29,30,31,32,33,34]. However, it is notable that the extent and range of available evidence-based literature on this topic remain limited. It is critical that future researchers explore effective mental health strategies that are tailored to the needs of children and adolescents, while balancing lifestyle transformation, which cannot be altered during pandemics (e.g., school closures, physical-distancing measures). Exploring effective channels regarding the development and delivery of evidenced-based age-appropriate educational materials is vital to future investigations on mental health. Moreover, it is essential to transcend the homogeneous tendency to develop and implement mental health interventions for children and adolescents. Amid this target population, gender, age, and educational needs vary, which the interventions that promote the overall mental health of children and adolescents must reflect. It is also important to address these needs with available healthcare resources and parental capacities and draw from the inherent strengths of children and adolescents.

### Limitations and Future Directions

While this rapid review was rigorous and applied a criterion for bias that allowed the evaluation of the methodology of the studies, there are limitations. Due to the short period of data collection there is a possibility of missing studies relevant to the mental health care of children and adolescents. Moreover, there is the possibility of publication bias i.e., only significant findings being published. One of the difficulties in comparing the studies in this review was the heterogeneity in the outcome measures. Therefore, we were not able to conduct meta-analyses. Moreover, the majority of the studies included in the review were based on online self-reports. The cross-sectional studies are useful in understanding the immediate or short-term impact apparent at a certain point of time. However, the limitations of these cross-sectional studies are that these studies cannot reach a conclusion about the long-term impact of pandemics. It is important to note that this rapid review could not prospectively examine changes in mental health outcomes as a result of this pandemic. Therefore, long-term impacts of the COVID-19 pandemic and elevated rates of mental health symptoms due to the pandemic could not be concluded in our study findings.

Our findings illustrate that pandemic or crisis situations could be associated with subsequent mental health problems in young people. With the objective of universal prevention and mental health promotion, it is important that children and adolescents should be informed about the pandemic situations. With the aim to increase children’s awareness about pandemics, it is crucial for parents, teachers and healthcare providers to communicate with young children in an age appropriate manner by using simple terminologies about COVID-19. Children need to be given fact-based information with the help of presentations and video material provided by authorized international organizations like WHO and UNICEF or government resources which have been tailor made especially for children. Adolescents are expected to have better knowledge about COVID 19 compared to young children. Therefore, communication has to be more open and non-directive with them.

Most of the adverse effects come from the school closure, isolation, limited physical activities, social distancing, and imposition of a restriction of liberty. Efforts should be made so that a consistent routine is followed by the child and adolescents during school closure, with enough opportunities to play, read, rest, and engage in physical activity. It is recommended that families play board games and engage in indoor sports activities with their children to avoid longer durations of video games. Parents should ensure that particularly the bedtime of a child is consistent. Similarly, excessive and irresponsible use of social media or internet gaming should be cautioned against. Negotiations with adolescents to limit their time and internet-based activities are recommended. More non-gadget related indoor activities and games are to be encouraged. There is also a need for evidence based elaborative strategies and a plan of action to cater to the mental health needs of children and adolescents during the period of pandemic.

Teachers have a role to play in the promotion of mental health among students. They can discuss what wellbeing is and how it is important for students. They can assist in teaching simple exercises, including deep breathing, muscle relaxation, distraction, and positive self -talk. Virtual workshops can be conducted in which “life skills” related to coping in stress can be in focus by using more practical examples. There is a need to enhance children’s and adolescents’ access to mental health services by using both face to face as well as digital platforms. There is a need for more virtual mental well-being programs in order to mitigate significant COVID-19 related mental health crises in children and adolescents for the duration of the current crisis and beyond. Finally, there is a pressing need for carrying out longitudinal and developmental studies to be able to apprehend multiple layers of dynamic determinants playing a role during this time of global crisis.

## 6. Conclusions

Although the rate of COVID-19 infection among young children and adolescents is low, the stress confronted by them poses their condition as highly vulnerable. The impact of the pandemic on children and adolescent’s mental health is inevitable. The findings from this knowledge synthesis identified the knowledge gaps and strengths and provide some evidence on effective interventions to prevent and manage mental health among adolescents and children during the midst of the pandemic. The real time analysis generated through our knowledge synthesis will be useful in developing evidence-based policy and practices during the current pandemic and will be transferable into future pandemic context. We hope that these knowledge synthesis findings will be useful for the global community and promote mental health of children and adolescents around the globe during the pandemic.

## Figures and Tables

**Table 1:** Inclusion and exclusion criteria.

Inclusion Criteria	Exclusion Criteria
*Types of studies:* Quantitative, qualitative, mixed methods studies, experimental (randomized (individually or cluster) and non-randomized controlled trials), observational studies with an internal comparison group (cohort-prospective and retrospective and case-control studies), Human studies *Types of participants:* studies that are conducted on school age children and adolescents (5 to 19 years), living in low-, middle- or high-income countries without any prior mental health disorder. *Type of exposure:* Recent COVID-19 or past pandemics (such as SARS-CoV-1, H1N1 influenza, equine influenza, Ebola, MERS-CoV etc.). Interventions delivered to school age children, adolescents and their families to improve mental health and decrease inclination towards substance abuse. *Types of outcomes:* Primary outcomes: rates of anxiety and depression and use of substance abuse. Secondary outcomes: fear of infection, frustration and boredom, fear of pandemic related uncertainty, fear of running out of basic supplies, and finances.	Not in English Studies that only included adults 18 years old or older Studies that did not report age Studies that included participants with prior mental health problems, those diagnosed with mental health disease, those who are homeless, and those with substance abuse issues.

**Table 2:** Characteristics of included studies (*n* = 18).

S #	Author and Year	Country	Study Design	Target Population	Total Participants	Exposure	Intervention	Outcomes
1	Decosimo et al., 2019 [17]	Liberia, West Africa	Pre and Post design	3–18 years	Treatment Group 1 (TG1), (*N* = 533) Treatment Group 2 (TG2), (*N* = 337) Total: 870 children	Ebola epidemic	Playing to live intervention including art therapy, play therapy, yoga therapy, support services, trained community facility, and cultural adaptation and community engagement program. TG1 received five months of the program. TG2 received three months of program	Statistically significant decrease in psychological stress symptoms over time before and after receiving the intervention in both treatment groups pre- to post-intervention and a significant difference in total symptoms over time.
2	Denis-Ramirez et al., 2017 [18]	Sierra Leone, West Africa	Qualitative study—Draw-and-write method	8–14 years	24 children	Ebola virus	N/A	Fear, Stigma
3	Kamara et al., 2017 [19]	Sierra Leone, West Africa	Prospective	0–17 years	27 children	Ebola virus	A nurse-led mental health and psychosocial support service via provision of psychotropic medication, psychological intervention, and social intervention	Depression Anxiety Grief Social problems. A nurse-led approach within a non-specialist setting was a successful model for delivering mental health and psychosocial support services during the Ebola outbreak in Sierra Leone.
	Taylor et al., 2008 [20]	Australia	Cross sectional—online survey	Under 16 years: 36 children 16–24 years: 224 participants	260 participants	Equine influenza	N/A	Psychological distress
5	Sprang et al., 2013 [21]	United States, Canada	Mixed-method approach survey, focus groups, and interviews	Children	586 Participants	H1N1	N/A	PTSD
6	Buzzi et al., 2020 [22]	Italy	Cross sectional—Online survey	13–19 years girls and boys	2064 adolescents	COVID-19	N/A	Consequences on psycho-social well-being
7	Chen et al., 2020 [23]	Guiyang, China	Cross sectional—Online survey	6–15 years	1036 Children Male 531 Female 505	COVID-19	N/A	Depression, Anxiety
8	Duan et al., 2020 [24]	China	Cross-sectional online survey design	7 to 18 year	3613 students 1812 males 1801 females	COVID-19	N/A	Anxiety Symptoms, Depressive symptoms, Coping mechanisms
9	Ellis et al., 2020 [25]	Ontario, Canada	Cross-sectional online survey design	14–18 years	1054 Participants	COVID-19	N/A	Stress, Loneliness, Depression
10	Liang et al., 2020 [26]	China	Cross-Sectional online survey	14–20 years	130 youths	COVID-19	N/A	PTSD, Coping styles, Mental health
11	Liu et al., 2020a [27]	Sichuan, China	Cross-sectional online survey	10–12 years 5–6 grade	209 primary school students Girls 93 Boys 116	COVID-19	N/A	Psychosomatic symptoms, Psychological distress
12	X. Liu et al., 2020b [28]	China	Cross-sectional online survey design	Below 18 years	34 participants	COVID-19	N/A	Anxiety, Depression, Psychological abnormalities
13	Oosterhoff et l., 2020 [29]	United States	Cross sectional—Online survey	13–18 years	683 adolescents	COVID-19	N/A	Anxiety symptoms. Depressive symptoms burdensomeness, belongingness
14	Saurabh et al., 2020 [30]	India	Cross sectional—survey questionnaire	9–18 years	252 children and adolescents	COVID-19	N/A	Worry, Helplessness, Fear
15	Tian et al., 2020 [31]	China	Cross-sectional online survey	Children under 18 years	22 participants	COVID-19	N/A	Psychological symptoms
16	Wang et al., 2020 [32]	China	Cross-sectional online survey design	12–21.4 years	344 participants	COVID-19	N/A	Stress, Anxiety, Depression
17	Zhou et al., 2020 [33]	China	Cross-sectional—online survey	12–18 years	8079 participants	COVID-19	N/A	Depression, Anxiety
18	Xie et al., 2020 [34]	Hubei, China	Cross-sectional online survey design	Children grade 2–6	1784 Students	COVID-19	N/A	Anxiety Symptoms, Depressive symptoms

## Data Availability

All data generated or analyzed during this study are included in this published article.

## Supplementary Materials

The following are available online at https://www.mdpi.com/article/10.3390/ijerph18073432/s1, File S1: Search Strategy.
