# Supplementary material for: Mental Health of Children and Adolescents Amidst COVID-19 and Past Pandemics: A Rapid Systematic Review
Source: Int J Environ Res Public Health. 2021 Mar 26;18(7):3432. doi: 10.3390/ijerph18073432 (PMC8038056; doi:10.3390/ijerph18073432)
Supplement: Supplementary file 1 [file ijerph-18-03432-s001.pdf]

File S1: Search strategy

Pubmed ALL <1990 to July 25, 2020>

(("2019 nCoV"[tiab] OR 2019nCoV[tiab] OR "2019 novel coronavirus"[tiab] OR "COVID 19"[tiab] OR COVID19[tiab] OR "new coronavirus"[tiab] OR "novel coronavirus"[tiab] OR "SARS CoV-2"[tiab] OR (Wuhan AND coronavirus)[tiab] OR "COVID 19"[tiab] OR "SARS-CoV"[tiab] OR "2019-nCoV"[tiab] OR "SARS-CoV-2"[tiab] OR "MERS"[tiab] OR "middle east respiratory syndrome coronavirus" [tiab] OR "MERS-CoV" [tiab] OR "SARS-CoV-1" [tiab] OR "SARS-CoV" [tiab] OR "ebola"[tiab] OR "EVD" [tiab] OR "ebola virus disease" [tiab] OR "H1N1" [tiab] OR "influenza" [tiab] OR "equine"[tiab] OR pandemic[tiab] OR Coronavirus [MeSH] OR Middle East Respiratory Syndrome Coronavirus [MeSH] OR SARS Virus [MeSH] OR COVID-19 [supplementary concept] OR severe acute respiratory syndrome coronavirus 2 [supplementary concept] OR Ebolavirus [MeSH] OR Hemorrhagic Fever, Ebola [MeSH] OR Influenza Pandemic, 1918-1919 [MeSH] OR Pandemics[MeSH]) AND ("mental health"[tiab] OR "anxiety"[tiab] OR "depression"[tiab] OR "fear"[tiab] OR "substance abuse"[tiab] OR "drug addict*"[tiab] OR "alcohol"[tiab] OR "smok*"[tiab] OR "weed"[tiab] OR "drug*"[tiab] OR heroin[tiab] OR drink[tiab] OR intoxicat*[tiab] OR opiate*[tiab] OR cocaine[tiab] OR methamphetamine[tiab] OR "crystal meth"[tiab] OR amphetamine*[tiab] OR cannabis[tiab] OR marijuana[tiab] OR marihunana[tiab] OR LSD[tiab] OR mushrooms[tiab] OR mephedrone OR khat[tiab] OR cathinone[tiab] OR ketamine[tiab] OR steroid*[tiab] OR "performance enhancing drug*"[tiab] OR gammahydroxybutrate OR GHB[tiab] OR "amyl nitrate" [tiab] OR Mental health [MeSH] OR depression[MeSH] OR Anxiety [MeSH] OR Behaviour, addictive [MeSH] OR alcohols[MeSH] OR ethacol[MeSH] OR heroin[MeSH] OR smoking[MeSH] OR alcoholic intoxication[MeSH] OR opiate alkoloids[MeSH] OR cocaine[MeSH] OR methamphetamine [MeSH] OR amphetamine[MeSH] OR Cannabis[MeSH] OR marijuana smoking[MeSH] OR marijuana abuse[MeSH] OR cathinone[MeSH] OR ketamine[MeSH] OR performance-enhancing substances[MeSH]) AND (Child[tiab] OR kid[tiab] OR "school-age"[tiab] OR adolesce*[tiab] OR "young" [tiab] OR teenage*[tiab] OR Child [MeSH] OR Adolescent [MeSH] OR young adult [MeSH])) AND ((intervention*[Title/Abstract] OR counsel?ing[Title/Abstract] OR psychotherap*[Title/Abstract] OR psycho-therap*[Title/Abstract] OR "cognitive behavior therapy"[Title/Abstract] OR "cognitive behaviour therapy"[Title/Abstract] OR CBT)[Title/Abstract])

Pubmed

Pandemics	Mental health	Children 5 to 19 years
"2019 nCoV"[tiab] OR 2019nCoV[tiab] OR "2019 novel coronavirus"[tiab] OR "COVID 19"[tiab] OR COVID19[tiab] OR "new coronavirus"[tiab] OR "novel coronavirus"[tiab] OR "SARS CoV-2"[tiab] OR (Wuhan AND coronavirus)[tiab] OR "COVID 19"[tiab] OR "SARS-CoV"[tiab] OR "2019-nCoV"[tiab] OR "SARS-CoV-2"[tiab] OR "MERS"[tiab] OR "middle east respiratory syndrome coronavirus" [tiab] OR "MERS-CoV" [tiab] OR "SARS-CoV-1" [tiab] OR "SARS-CoV" [tiab] OR "ebola"[tiab] OR "EVD" [tiab] OR "ebola virus disease" [tiab] OR "H1N1" [tiab] OR "influenza" [tiab] OR "equine"[tiab] OR pandemic[tiab] OR	"mental health"[tiab] OR "anxiety"[tiab] OR "depression"[tiab] OR "fear"[tiab] OR "substance abuse"[tiab] OR "drug addict*"[tiab] OR "alcohol"[tiab] OR "smok*"[tiab] OR "weed"[tiab] OR "drug*"[tiab] OR heroin[tiab] OR drink[tiab] OR intoxicat*[tiab] OR opiate*[tiab] OR cocaine[tiab] OR methamphetamine[tiab] OR "crystal meth"[tiab] OR amphetamine*[tiab] OR cannabis[tiab] OR marijuana[tiab] OR marihunana[tiab] OR LSD[tiab] OR mushrooms[tiab] OR mephedrone OR khat[tiab] OR cathinone[tiab] OR ketamine[tiab] OR steroid*[tiab] OR "performance enhancing drug*"[tiab] OR gammahydroxybutrate OR GHB[tiab] OR "amyl nitrate" [tiab] OR<br><br>Mental health [MeSH] OR depression[MeSH] OR Anxiety [MeSH] OR Behaviour, addictive [MeSH] OR alcohols[MeSH] OR ethacol[MeSH] OR heroin[MeSH] OR smoking[MeSH] OR alcoholic intoxication[MeSH] OR opiate alkoloids[MeSH] OR cocaine[MeSH] OR methamphetamine [MeSH]	Child[tiab] OR kid[tiab] OR "school-age"[tiab] OR adolesce*[tiab] OR "young" [tiab] OR teenage*[tiab] OR<br><br>Child [MeSH] OR Adolescent [MeSH] OR young adult [MeSH] OR

Coronavirus [MeSH] OR Middle East Respiratory Syndrome Coronavirus [MeSH] OR SARS Virus [MeSH] OR COVID-19 [MeSH] OR severe acute respiratory syndrome coronavirus 2 [MeSH] OR Ebolavirus [MeSH] OR Hemorrhagic Fever, Ebola [MeSH] OR Influenza Pandemic, 1918-1919 [MeSH] OR Pandemics[MeSH]	OR amphetamine[MeSH] OR Cannabis[MeSH] OR marijuana smoking[MeSH] OR marijuana abuse[MeSH] OR cathinone[MeSH] OR ketamine[MeSH] OR performance-enhancing substances[MeSH]	

("2019 nCoV"[tiab] OR 2019nCoV[tiab] OR "2019 novel coronavirus"[tiab] OR "COVID 19"[tiab] OR COVID19[tiab] OR "new coronavirus"[tiab] OR "novel coronavirus"[tiab] OR "SARS CoV-2"[tiab] OR (Wuhan AND coronavirus)[tiab] OR "COVID 19"[tiab] OR "SARS-CoV"[tiab] OR "2019-nCoV"[tiab] OR "SARS-CoV-2"[tiab] OR "MERS"[tiab] OR "middle east respiratory syndrome coronavirus" [tiab] OR "MERS-CoV" [tiab] OR "SARS-CoV-1" [tiab] OR "SARS-CoV" [tiab] OR "ebola"[tiab] OR "EVD" [tiab] OR "ebola virus disease" [tiab] OR "H1N1" [tiab] OR "influenza" [tiab] OR "equine"[tiab] OR pandemic[tiab] OR Coronavirus [MeSH] OR Middle East Respiratory Syndrome Coronavirus [MeSH] OR SARS Virus [MeSH] OR COVID-19 [MeSH] OR severe acute respiratory syndrome coronavirus 2 [MeSH] OR Ebolavirus [MeSH] OR Hemorrhagic Fever, Ebola [MeSH] OR Influenza Pandemic, 1918-1919 [MeSH] OR Pandemics[MeSH]) **AND** ("mental health"[tiab] OR "anxiety"[tiab] OR "depression"[tiab] OR "fear"[tiab] OR "substance abuse"[tiab] OR "drug addict*"[tiab] OR "alcohol"[tiab] OR "smok*"[tiab] OR "weed"[tiab] OR "drug*"[tiab] OR heroin[tiab] OR drink[tiab] OR intoxicat*[tiab] OR opiate*[tiab] OR cocaine[tiab] OR methamphetamine[tiab] OR "crystal meth"[tiab] OR amphetamine*[tiab] OR cannabis[tiab] OR marijuana[tiab] OR marihunana[tiab] OR LSD[tiab] OR mushrooms[tiab] OR mephedrone OR khat[tiab] OR cathinone[tiab] OR ketamine[tiab] OR steroid*[tiab] OR "performance enhancing drug*"[tiab] OR gammahydroxybutrate OR GHB[tiab] OR "amyl nitrate" [tiab] OR Mental health [MeSH] OR depression[MeSH] OR Anxiety [MeSH] OR Behaviour, addictive [MeSH] OR alcohols[MeSH] OR ethacol[MeSH] OR heroin[MeSH] OR smoking[MeSH] OR alcoholic intoxication[MeSH] OR opiate alkoloids[MeSH] OR cocaine[MeSH] OR methamphetamine [MeSH] OR amphetamine[MeSH] OR Cannabis[MeSH] OR marijuana smoking[MeSH] OR marijuana abuse[MeSH] OR cathinone[MeSH] OR ketamine[MeSH] OR performance-enhancing substances[MeSH]) **AND** (Child[tiab] OR kid[tiab] OR "school-age"[tiab] OR adolesce*[tiab] OR "young" [tiab] OR teenage*[tiab] OR Child [MeSH] OR Adolescent [MeSH] OR young adult [MeSH])

Embase <1990 to 2020 July 25>

Embase

("2019 nCoV":ti,ab OR #2019 nCoV:ti,ab OR "2019 novel coronavirus":ti,ab OR "COVID 19":ti,ab OR COVID19:ti,ab OR "new coronavirus":ti,ab OR "novel coronavirus":ti,ab OR "SARS CoV-2":ti,ab OR (Wuhan AND coronavirus):ti,ab OR "COVID 19":ti,ab OR SARS-CoV:ti,ab OR 2019-nCoV:ti,ab OR SARS-CoV-2:ti,ab OR MERS:ti,ab OR "middle east respiratory syndrome coronavirus":ti,ab OR MERS-CoV:ti,ab OR SARS-CoV-1:ti,ab OR SARS-CoV:ti,ab OR ebola:ti,ab OR EVD:ti,ab OR "ebola virus disease":ti,ab OR H1N1:ti,ab OR influenza:ti,ab OR equine:ti,ab OR pandemic:ti,ab OR 'Coronavirus '/exp OR 'Middle East Respiratory Syndrome Coronavirus '/exp OR 'SARS Virus '/exp OR 'COVID-19 '/exp OR 'severe acute respiratory syndrome coronavirus '/exp OR 'Ebolavirus '/exp OR 'Hemorrhagic Fever, Ebola '/exp OR 'Influenza Pandemic, 1918-'/exp OR 'Pandemics'/exp) AND ("mental health":ti,ab OR anxiety:ti,ab OR depression:ti,ab OR fear:ti,ab OR "substance abuse":ti,ab OR "drug addict*":ti,ab OR alcohol:ti,ab OR smok*:ti,ab OR weed:ti,ab OR drug*:ti,ab OR heroin:ti,ab OR drink:ti,ab OR intoxicat*:ti,ab OR opiate*:ti,ab OR cocaine:ti,ab OR methamphetamine:ti,ab OR "crystal meth":ti,ab OR amphetamine*:ti,ab OR cannabis:ti,ab OR marijuana:ti,ab OR marihunana:ti,ab OR LSD:ti,ab OR mushrooms:ti,ab OR mephedrone OR khat:ti,ab OR cathinone:ti,ab OR ketamine:ti,ab OR steroid*:ti,ab OR "performance enhancing drug*":ti,ab OR gammahydroxybutrate OR GHB:ti,ab OR "amyl nitrate":ti,ab OR 'Mental health '/exp OR 'depression'/exp OR 'Anxiety '/exp OR 'Behaviour, addictive '/exp OR 'alcohols'/exp OR 'ethacol'/exp OR 'heroin'/exp OR 'smoking'/exp OR 'alcoholic intoxication'/exp OR 'opiate alkoloids'/exp OR 'cocaine'/exp OR 'methamphetamine '/exp OR 'amphetamine'/exp OR 'Cannabis'/exp OR 'marijuana smoking'/exp OR 'marijuana abuse'/exp OR 'cathinone'/exp

OR 'ketamine'/exp OR 'performance-enhancing substances'/exp) AND (Child:ti,ab OR kid:ti,ab OR school-age:ti,ab OR adolesce*:ti,ab OR young:ti,ab OR teenage*:ti,ab OR 'Child '/exp OR 'Adolescent '/exp OR 'young adult '/exp)

Web of Science

("2019 nCoV" OR #2019 nCoV OR "2019 novel coronavirus" OR "COVID 19" OR COVID19 OR "new coronavirus" OR "novel coronavirus" OR "SARS CoV-2" OR (Wuhan AND coronavirus) OR "COVID 19" OR SARS-CoV OR 2019-nCoV OR SARS-CoV-2 OR MERS OR "middle east respiratory syndrome coronavirus" OR MERS-CoV OR SARS-CoV-1 OR SARS-CoV OR ebola OR EVD OR "ebola virus disease" OR H1N1 OR influenza OR equine OR pandemic OR Coronavirus OR "Middle East Respiratory Syndrome Coronavirus" OR "SARS Virus" OR COVID-19 OR "severe acute respiratory syndrome coronavirus" OR Ebolavirus OR "Hemorrhagic Fever, Ebola" OR "Influenza Pandemic, 1918-" OR Pandemics) AND ("mental health" OR anxiety OR depression OR fear OR "substance abuse" OR "drug addict*" OR alcohol OR smok* OR weed OR drug* OR heroin OR drink OR intoxicat* OR opiate* OR cocaine OR methamphetamine OR "crystal meth" OR amphetamine* OR cannabis OR marijuana OR marihunana OR LSD OR mushrooms OR mephedrone OR khat OR cathinone OR ketamine OR steroid* OR "performance enhancing drug*" OR gammahydroxybutrate OR GHB OR "amyl nitrate" OR "Mental health" OR depression OR Anxiety OR "Behaviour, addictive" OR alcohols OR ethacol OR heroin OR smoking OR "alcoholic intoxication" OR "opiate alkoloids" OR cocaine OR methamphetamine OR amphetamine OR Cannabis OR "marijuana smoking" OR "marijuana abuse" OR cathinone OR ketamine OR "performance-enhancing substances") AND (Child OR kid OR school-age OR adolesce* OR young OR teenage* OR Child OR Adolescent OR "young adult")

Central Cochrane Library

("2019 nCoV":ti,ab OR #2019 nCoV:ti,ab OR "2019 novel coronavirus":ti,ab OR "COVID 19":ti,ab OR COVID19:ti,ab OR "new coronavirus":ti,ab OR "novel coronavirus":ti,ab OR "SARS CoV-2":ti,ab OR (Wuhan AND coronavirus):ti,ab OR "COVID 19":ti,ab OR SARS-CoV:ti,ab OR 2019-nCoV:ti,ab OR SARS-CoV-2:ti,ab OR MERS:ti,ab OR "middle east respiratory syndrome coronavirus":ti,ab OR MERS-CoV:ti,ab OR SARS-CoV-1:ti,ab OR SARS-CoV:ti,ab OR ebola:ti,ab OR EVD:ti,ab OR "ebola virus disease":ti,ab OR H1N1:ti,ab OR influenza:ti,ab OR equine:ti,ab OR pandemic:ti,ab OR [mh Coronavirus] OR [mh "Middle East Respiratory Syndrome Coronavirus"] OR [mh "SARS Virus"] OR [mh COVID-19] OR [mh "severe acute respiratory syndrome coronavirus"] OR [mh Ebolavirus] OR [mh "Hemorrhagic Fever, Ebola"] OR [mh "Influenza Pandemic, 1918-"] OR [mh Pandemics]) AND ("mental health":ti,ab OR anxiety:ti,ab OR depression:ti,ab OR fear:ti,ab OR "substance abuse":ti,ab OR "drug addict*":ti,ab OR alcohol:ti,ab OR smok*:ti,ab OR weed:ti,ab OR drug*:ti,ab OR heroin:ti,ab OR drink:ti,ab OR intoxicat*:ti,ab OR opiate*:ti,ab OR cocaine:ti,ab OR methamphetamine:ti,ab OR "crystal meth":ti,ab OR amphetamine*:ti,ab OR cannabis:ti,ab OR marijuana:ti,ab OR marihunana:ti,ab OR LSD:ti,ab OR mushrooms:ti,ab OR
Mephedrone OR khat:ti,ab OR cathinone:ti,ab OR ketamine:ti,ab OR steroid*:ti,ab OR "performance enhancing drug*":ti,ab OR Gammahydroxybutrate OR GHB:ti,ab OR "amyl nitrate":ti,ab OR [mh "Mental health"] OR [mh depression] OR [mh Anxiety] OR [mh "Behaviour, addictive"] OR [mh alcohols] OR [mh ethacol] OR [mh heroin] OR [mh smoking] OR [mh "alcoholic intoxication"] OR [mh "opiate alkoloids"] OR [mh cocaine] OR [mh methamphetamine] OR [mh amphetamine] OR [mh Cannabis] OR [mh "marijuana smoking"] OR [mh "marijuana abuse"] OR [mh

cathinone] OR [mh ketamine] OR [mh "performance-enhancing substances"]) AND (Child:ti,ab OR kid:ti,ab OR school-age:ti,ab OR adolesce*:ti,ab OR young:ti,ab OR teenage*:ti,ab OR [mh Child] OR [mh Adolescent] OR [mh "young adult"])

CINAHL

(TI "2019 nCoV" OR AB "2019 nCoV" OR S2019 TI nCoV OR AB nCoV OR TI "2019 novel coronavirus" OR AB "2019 novel coronavirus" OR TI "COVID 19" OR AB "COVID 19" OR TI COVID19 OR AB COVID19 OR TI "new coronavirus" OR AB "new coronavirus" OR TI "novel coronavirus" OR AB "novel coronavirus" OR TI "SARS CoV-2" OR AB "SARS CoV-2" OR (TI Wuhan OR AB Wuhan AND TI coronavirus OR AB coronavirus) OR TI "COVID 19" OR AB "COVID 19" OR TI SARS-CoV OR AB SARS-CoV OR TI 2019-nCoV OR AB 2019-nCoV OR TI SARS-CoV-2 OR AB SARS-CoV-2 OR TI MERS OR AB MERS OR TI "middle east respiratory syndrome coronavirus" OR AB "middle east respiratory syndrome coronavirus" OR TI MERS-CoV OR AB MERS-CoV OR TI SARS-CoV-1 OR AB SARS-CoV-1 OR TI SARS-CoV OR AB SARS-CoV OR TI ebola OR AB ebola OR TI EVD OR AB EVD OR TI "ebola virus disease" OR AB "ebola virus disease" OR TI H1N1 OR AB H1N1 OR TI influenza OR AB influenza OR TI equine OR AB equine OR TI pandemic OR AB pandemic OR (MH "Coronavirus +") OR (MH "Middle East Respiratory Syndrome Coronavirus +") OR (MH "SARS Virus +") OR (MH "COVID-19 +") OR (MH "severe acute respiratory syndrome coronavirus +") OR (MH "Ebolavirus +") OR (MH "Hemorrhagic Fever, Ebola +") OR (MH "Influenza Pandemic, 1918-+") OR (MH "Pandemics+")) AND (TI "mental health" OR AB "mental health" OR TI anxiety OR AB anxiety OR TI depression OR AB depression OR TI fear OR AB fear OR TI "substance abuse" OR AB "substance abuse" OR TI "drug addict*" OR AB "drug addict*" OR TI alcohol OR AB alcohol OR TI smok* OR AB smok* OR TI weed OR AB weed OR TI drug* OR AB drug* OR TI heroin OR AB heroin OR TI drink OR AB drink OR TI intoxicat* OR AB intoxicat* OR TI opiate* OR AB opiate* OR TI cocaine OR AB cocaine OR TI methamphetamine OR AB methamphetamine OR TI "crystal meth" OR AB "crystal meth" OR TI amphetamine* OR AB amphetamine* OR TI cannabis OR AB cannabis OR TI marijuana OR AB marijuana OR TI marihunana OR AB marihunana OR TI LSD OR AB LSD OR TI mushrooms OR AB mushrooms OR mephedrone OR TI khat OR AB khat OR TI cathinone OR AB cathinone OR TI ketamine OR AB ketamine OR TI steroid* OR AB steroid* OR TI "performance enhancing drug*" OR AB "performance enhancing drug*" OR Gammahydroxybutrate OR TI GHB OR AB GHB OR TI "amyl nitrate" OR AB "amyl nitrate" OR (MH "Mental health +") OR (MH "depression+") OR (MH "Anxiety +") OR (MH "Behaviour, addictive +") OR (MH "alcohols+") OR (MH "ethacol+") OR (MH "heroin+") OR (MH "smoking+") OR (MH "alcoholic intoxication+") OR (MH "opiate alkoloids+") OR (MH "cocaine+") OR (MH "methamphetamine +") OR (MH "amphetamine+") OR (MH "Cannabis+") OR (MH "marijuana smoking+") OR (MH "marijuana abuse+") OR (MH "cathinone+") OR (MH "ketamine+") OR (MH "performance-enhancing substances+")) AND (TI Child OR AB Child OR TI kid OR AB kid OR TI school-age OR AB school-age OR TI adolesce* OR AB adolesce* OR TI young OR AB young OR TI teenage* OR AB teenage* OR (MH "Child +") OR (MH "Adolescent +") OR (MH "young adult +"))

PsycInfo

("2019 nCoV".ti,ab OR #2019 nCoV.ti,ab OR "2019 novel coronavirus".ti,ab OR "COVID 19".ti,ab OR COVID19.ti,ab OR "new coronavirus".ti,ab OR "novel coronavirus".ti,ab OR "SARS CoV-2".ti,ab OR (Wuhan.ti,ab AND coronavirus.ti,ab) OR "COVID 19".ti,ab OR SARS-CoV.ti,ab OR 2019-nCoV.ti,ab OR SARS-CoV-2.ti,ab OR MERS.ti,ab OR "middle east respiratory syndrome coronavirus".ti,ab OR MERS-CoV.ti,ab OR SARS-CoV-1.ti,ab

OR SARS-CoV.ti,ab OR ebola.ti,ab OR EVD.ti,ab OR "ebola virus disease".ti,ab OR H1N1.ti,ab OR influenza.ti,ab OR equine.ti,ab OR pandemic.ti,ab OR Coronavirus OR "Middle East Respiratory Syndrome Coronavirus" OR "SARS Virus" OR COVID-19 OR "severe acute respiratory syndrome coronavirus" OR Ebolavirus OR "Hemorrhagic Fever, Ebola" OR "Influenza Pandemic, 1918-" OR Pandemics) AND ("mental health".ti,ab OR anxiety.ti,ab OR depression.ti,ab OR fear.ti,ab OR "substance abuse".ti,ab OR "drug addict*".ti,ab OR alcohol.ti,ab OR smok*.ti,ab OR weed.ti,ab OR drug*.ti,ab OR heroin.ti,ab OR drink.ti,ab OR intoxicat*.ti,ab OR opiate*.ti,ab OR cocaine.ti,ab OR methamphetamine.ti,ab OR "crystal meth".ti,ab OR amphetamine*.ti,ab OR cannabis.ti,ab OR marijuana.ti,ab OR marihunana.ti,ab OR LSD.ti,ab OR mushrooms.ti,ab OR mephedrone OR khat.ti,ab OR cathinone.ti,ab OR ketamine.ti,ab OR steroid*.ti,ab OR "performance enhancing drug*".ti,ab OR gammahydroxybutrate OR GHB.ti,ab OR "amyl nitrate".ti,ab OR "Mental health" OR depression OR Anxiety OR "Behaviour, addictive" OR alcohols OR ethacol OR heroin OR smoking OR "alcoholic intoxication" OR "opiate alkoloids" OR cocaine OR methamphetamine OR amphetamine OR Cannabis OR "marijuana smoking" OR "marijuana abuse" OR cathinone OR ketamine OR "performance-enhancing substances") AND (Child.ti,ab OR kid.ti,ab OR school-age.ti,ab OR adolesce*.ti,ab OR young.ti,ab OR teenage*.ti,ab OR Child OR Adolescent OR "young adult")

Hand search WHO COVID-19 database and MEdRxiv
